# Family-Related Opinions and Stressful Situations Associated with Psychological Distress in Women Undergoing Infertility Treatment

**DOI:** 10.3390/ijerph110909068

**Published:** 2014-09-02

**Authors:** Jiro Takaki, Yuri Hibino

**Affiliations:** 1 Department of Public Health and Occupational Medicine, Mie University Graduate School of Medicine, 2-174 Edobashi, Tsu, Mie 514-8507, Japan; 2 Department of Environmental and Preventive Medicine, Graduate School of Medical Science, Kanazawa University, Kakumamachi, Kanazawa, Ishikawa 920-1192, Japan; E-Mail: hibino@staff.kanazawa-u.ac.jp

**Keywords:** infertility, psychological distress, stress

## Abstract

The purpose of this study is to investigate how family-related opinions and stressful situations are related to psychological distress in women undergoing infertility treatment. The subjects in this cross-sectional study were recruited from female patients undergoing infertility treatment (*n* = 2540) at 70 infertility treatment institutions in Japan. Because of non-participation or missing data, the number of subjects included in the analysis was 635 (response rate, 25.0%). The family-related opinions and stressful situations were evaluated using the original questions. Psychological distress was assessed using a self-report measure, the Kessler Six-question Psychological Distress Scale (K6). The K6 scores of the following participants were significantly (*p* < 0.05) and independently high: those with more frequent miscarriage/stillbirth/abortions, those with repeated miscarriages as the cause of infertility, those with infertility of unknown causes, those living with no child, those having a low joint income with their partner, those with the opinion that “women should devote themselves to their household duties” those who had considered stopping treatment, those without the opinion that “married life without children is favorable” and those who had experienced stressful situations such as inadequate explanation by doctors, frustration of multiple failed attempts, differences of opinion with the partner, and lack of knowledge regarding when to stop treatment. Family-related opinions and stressful situations associated with psychological distress in women undergoing infertility treatment are outlined. The results of this study may contribute to the prevention of and care for psychological distress in female patients undergoing infertility treatment.

## 1. Introduction

The clinical definition of infertility as used by the World Health Organization (WHO) is “a disease of the reproductive system defined by a failure to achieve clinical pregnancy after 12 months or more of regular unprotected sexual intercourse” [1], whereas the WHO’s epidemiologic definition is “women of reproductive age at risk of becoming pregnant who report unsuccessful attempts at achieving pregnancy for more than 2 years” [2]. When infertility was defined as the absence of a live birth for women who desire a child and who have been in a union for at least 5 years during which they have not used any contraceptive, the absolute number of couples affected by infertility increased from 42.0 million (95% uncertainty interval, 39.6–44.8 million) in 1990 to 48.5 million (95% uncertainty interval, 45.0–52.6 million) in 2010 [3].

The world’s first *in vitro* fertilization (IVF) baby was born in 1978 [4]. Since then, many infertile patients have had children using assisted reproductive technology (ART). The International Committee for Monitoring Assisted Reproduction Technology of the European Society of Human Reproduction and Embryology estimated that up to 2011, approximately 5 million children had been born using ART. In Japan, the first IVF birth occurred in 1983, and approximately 29,000 births (2.7% of all births) were achieved by IVF in 2010 [5], indicating that many infertile patients have benefited from ART.

In addition to the psychological impact of infertility, infertility treatment places physical, economic, and emotional burdens [6,7]. The unfulfilled desire to have a child and the consequent threat of permanent infertility have been shown to be related to increased levels of anxiety and depression during IVF treatment [8]. In a recent study, the prevalence of clinical depression ranges from 26% to 44% in women undergoing IVF treatment [9].

In a recent retrospective cohort study of 98,320 Danish women [10], the incidence rate for hospitalization for all mental disorders was 393 and 353 cases per 100,000 person-years among women who did not give birth and those who did give birth after an infertility evaluation, respectively. The risk of hospitalization for affective disorders was lower among women who did not give birth after the infertility evaluation than among those who did give birth [10]. However, most mood disorders can be treated on an out-patient basis. Hospitalization is often indicated for patients at risk of hurting themselves or others, patients who cannot take care of themselves, or patients who need management and monitoring of complicated or novel psychopharmacological regimens [11]. During infertility treatments, women’s partners often take care of the women, and the women usually worry about the influence of psychopharmacotherapy on their pregnancies. These women may consider beginning psychopharmacotherapy after giving birth.

Some previous studies have suggested that depression worsened in infertile women as age and the duration of infertility increased [12,13,14,15,16,17,18,19,20]; however, such associations were not observed in other previous studies [9,21,22,23]. An earlier study suggested that having a job was inversely associated with depression in infertile women [12]. Another study suggested that infertile women were at a higher risk of developing psychiatric disorders if they were housewives rather than working women [24]. In the present study, we hypothesized that apart from demographic factors such as age and occupational status, family-related opinions (opinions about working women; opinions toward infertility treatment, life, and adoption) and stressful situations were related to psychological distress in female patients undergoing infertility treatment. The purpose of this study is to investigate how family-related opinions and stressful situations are related to psychological distress in women undergoing infertility treatment.

## 2. Methods

### 2.1. Subjects

The subjects of this cross-sectional study were female patients undergoing infertility treatments (*n* = 2540) at the 70 infertility treatment institutions in Japan. From February 2013 to April 2013, questionnaires were mailed to the institutions, and the institutional staff distributed them to the patients. The purpose and procedure of the survey were explained to the participants in the documents. Written informed consent was obtained from all participants and there was no compensation for participation. By April 2013, a total of 740 questionnaires were returned to us by mail (response rate, 29.1%). Of these, 105 subjects were excluded because of missing data, and a total of 635 subjects (final response rate, 25.0%) were included for analysis. This study was approved by the Institutional Review Board of the Graduate School of Medical Science, Kanazawa University.

### 2.2. Measures

The participants completed a self-administered questionnaire that included background information such as age, length of marriage, number of children in the household, annual income, employment status, number of miscarriages/stillbirths/abortions, length of infertility treatment, and causes of infertility (multiple answers). For annual income, we used the Japanese yen; the exchange rate was 100 Japanese yen to 1 United States dollar (April 2013). The questionnaire also included measures to evaluate family-related opinions, stressful situations, and psychological distress.

As the preliminary step, we interviewed infertile women about their family-related opinions and stressful situations using open questioning. We then drafted the original questions shown in Table 1 that evaluated family-related opinions and stressful situations in infertile women. Regarding the Opinion questions (c) and (d) and all the Situation questions, participants who answered “1” or “2” were categorized as ”Yes”; participants who answered “3”, “4”, or “5” were categorized as “No”. Both English and Japanese versions of the Family-related opinions and Stressful situations questions were prepared. The original items in Japanese were translated into English by two independent native English speakers and then back-translated into Japanese. The back-translation was then checked by the authors.

**Table 1:** Original questionnaire.

Opinion					
**(a) Please circle the statement that best describes what you think about “working women”.**					
1. Women should try to reconcile working and housekeeping/bringing up children.					
2. The woman's job should not interfere with her household duties and child-care responsibilities.					
3. Women should devote themselves to their household duties.					
4. Other ( )					
**(b) Have you considered stopping treatment?** 1.Yes 2. No					
**(c) Please circle the number that best represents your reaction to married life without children if it becomes clear that your eggs do not work.**					
1. Very favorable 2. Somewhat favorable 3. Undecided 4. Somewhat unfavorable 5. Very unfavorable					
**(d) Please circle the number that best represents your reaction to the statement that an adoptive relationship would make you insecure.**					
1. Strongly agree 2. Somewhat agree 3. Undecided 4. Somewhat disagree 5. Strongly disagree					
**Situation**					
**Please circle the number that best represents your stressful situation.**					
1. Strongly agree 2. Somewhat agree 3. Undecided 4. Somewhat disagree 5. Strongly disagree					
(**a**) The high cost of treatments	1	2	3	4	5
(**b**) Insufficient explanation by doctors	1	2	3	4	5
(**c**) The frustration of multiple failed attempts	1	2	3	4	5
(**d**) Differences of opinion with my partner	1	2	3	4	5
(**e**) Strong peer and family pressure	1	2	3	4	5
(**f**) Feelings of guilt toward my partner	1	2	3	4	5
(**g**) No idea of when to stop the treatment	1	2	3	4	5
(**h**) The physical stresses of the treatment	1	2	3	4	5
(**i**) Worries about the future health effects of the treatment	1	2	3	4	5

To evaluate non-specific psychological distress, we used a self-report measure, the Kessler Six-question Psychological Distress Scale (K6) [25]. The K6 was developed for the redesigned United States National Health Interview Survey (NHIS). The K6 helps assess how frequently respondents experience symptoms of psychological distress (e.g., feeling so sad that nothing can produce cheer) during the previous 30 days [25]. The responses are recorded using a five-category scale (4 = all the time, 3 = most of the time, 2 = some of the time, and 1 = none of the time), yielding a score range of 0–24 [25]. The K6 has been shown to be a sensitive screen for mental disorders that can be diagnosed using the Structured Clinical Interview for the Diagnostic and Statistical Manual of Mental Disorders (4th edition) which was developed by the American Psychiatric Association (DSM-IV/SCID) through surveys carried out in the United States [25]. The K6 has been translated into Japanese, and it has been shown to have acceptable reliability and validity for measuring levels of psychological distress [26]. A cut-off point of >8 on the K6 measures psychological distress in the Japanese population with 77.8% sensitivity and 86.4% specificity [27]. In previous studies [28,29], the K6 has been found to be an effective screening method for psychological distress, with results that are as reliable as those of other assessments such as the Depression and Suicide Screen (DSS), and the Center for Epidemiologic Studies Depression Scale (CES-D). The K6 has also been used to predict suicidal behavior during the past year [30].

### 2.3. Statistical Analyses

Crude associations for the background information, family-related opinions, and stressful situations with the K6 scores were assessed using linear regression analyses. Associations that were mutually adjusted for all the variables were also assessed. Unstandardized regression coefficients demonstrated differences in the mean K6 scores from the reference group for the categorical variables and changes in the K6 scores per one-unit increase in the continuous variables. All *p* values were two-tailed, and *p* < 0.05 was considered as the threshold for significance. All statistical analyses were performed using SPSS version 20 (IBM Japan, Chuo-ku, Tokyo, Japan).

## 3. Results

Distribution of the K6 score was as follows: mean = 7.5; standard deviation = 5.6; and range, 0–24. If a cut-off point of >8 on the K6 scores was used, 232 participants (36.5%) were regarded as having psychological distress. Table 2 shows the subjects’ characteristics and the associations with the K6 scores. In the crude analyses, the number of miscarriages/stillbirths/abortions were significantly positively associated with the K6 scores, and the K6 scores of the following participants were significantly high: those whose cause of infertility was precocious menopause, those whose cause of infertility was repeated miscarriage, those whose cause of infertility was unknown, those living with no child, those having an annual joint income with their partner of <40,000 dollars and of ≥40,000 and <60,000 dollars, those who had considered stopping treatment, those not of the opinion that “married life without children is favorable”, those of the opinion that “an adoptive relationship would make me insecure”, and those who had experienced stressful situations such as high costs of treatment, insufficient explanations by doctors, frustration of multiple failed fertilization attempts, differences of opinion with the partner, strong peer and family pressure, feelings of guilt toward the partner, lack of knowledge regarding when to stop treatment, physical stress of the treatment, and worry about future health effects of the treatment.

In the multivariable analyses mutually adjusted for all variables, the number of miscarriage/stillbirth/abortion were significantly positively associated with the K6 scores, and the K6 scores of the following participants were significantly and independently high: those whose cause of infertility was repeated miscarriage, those whose cause of infertility was unknown, those living with no child, those having an annual joint income with their partner of <40,000 dollars and of ≥40,000 and <60,000 dollars, those who had considered stopping treatment, those not of the opinion that “married life without children is favorable”, and those who had experienced stressful situations such as insufficient explanation by doctors, frustration of multiple failed attempts, differences of opinion with the partner, and lack of knowledge regarding when to stop the treatment.

## 4. Discussion

In the crude analyses, psychological distress was found to be associated with precocious menopause as the cause of infertility, the opinion that “an adoptive relationship would make me insecure”, and the experience of stressful situations such as high cost of treatment, strong peer and family pressure, feelings of guilt toward the partner, lack of knowledge regarding when to stop treatment, physical stress of the treatment, and worries about future health effects of the treatment; however, these associations were not observed in the multivariable analyses mutually adjusted for all variables. Such associations might be mediated or confounded by the other associations investigated.

In the multivariable analyses mutually adjusted for all variables, psychological distress was independently associated with the factors of more frequent miscarriages/stillbirths/abortions, repeated miscarriages as the cause of infertility, unknown causes of infertility, living with no child, an annual joint income with their partner of <40,000 dollars and of ≥40,000 and <60,000 dollars, consideration of stopping of treatment, not being of the opinion that “married life without children is favorable”, and experiencing stressful situations such as insufficient explanation by doctors, frustration of multiple failed attempts, differences of opinion with the partner, and lack of knowledge regarding when to stop the treatment. Identification of patients that are at risk for psychological distress can be important for prevention and care in women undergoing infertility treatment. Preparatory psychosocial counseling for medically-assisted reproduction or cognitive-behavioral therapy (relaxation, guided imagery, and stress management) might be useful for them [31,32]. Government financial support might prevent or reduce psychological distress associated with low income. Enhancement of communication among the infertile couple and the patient care team might prevent or reduce psychological distress associated with insufficient explanation by doctors, differences of opinion with the partner, and lack of knowledge regarding when to stop the treatment.

The strength of this study was that a validated instrument was used to measure psychological distress (K6). Further, we used a relatively large sample size compared with previous studies on psychological distress in infertile women [6,8,9,12,13,14,15,16,17,18,19,20,21,22,23,24,33]. However, the limitations of our study must also be noted. First, all measurements were self-reported; thus, more objective measurements are needed in future studies. Second, the use of a cross-sectional design did not allow us to determine causality in our results; future experimental intervention research is needed.

**Table 2:** Participant characteristics and results of linear regression analyses for the Kessler Six-question Psychological Distress Scale (K6) scores (*n* = 635).

Independent Variable	Summary Statistics		Crude			All the Variables Mutually Adjusted		
			B	SE	*p*	B	SE	*p*
Age (years)	Mean = 36.4		−0.06	0.05	0.264	−0.03	0.06	0.586
	SD = 4.5							
	Range: 25-49							
Marriage length (years)	Mean = 5.5		−0.10	0.06	0.097	−0.03	0.07	0.666
	SD = 3.6							
	Range: 0.3-20							
Miscarriage/stillbirth/abortion (times)	Median = 0		**0.58**	**0.23**	**0.012**	**0.48**	**0.22**	**0.034**
	Range: 0-6							
Total infertility treatment length (years)	Median = 2		0.05	0.09	0.589	−0.05	0.11	0.654
	Range: 0-20							
	**N**	**%**						
Cause of infertility (multiple answers)								
Uterine or cervical problem								
No	425	66.9	Referent			Referent		
Yes	210	33.1	0.51	0.47	0.284	−0.01	0.45	0.986
Fallopian tube problem								
No	500	78.7	Referent			Referent		
Yes	135	21.3	0.50	0.55	0.363	0.69	0.51	0.177
Ovum or ovarian problem								
No	477	75.1	Referent			Referent		
Yes	158	24.9	0.65	0.52	0.207	0.18	0.52	0.730
Precocious menopause								
No	612	96.4	**Referent**			Referent		
Yes	23	3.6	**2.69**	**1.19**	**0.024**	1.52	1.13	0.181
Age-related infertility								
No	339	53.4	Referent			Referent		
Yes	296	46.6	0.31	0.45	0.483	0.65	0.52	0.211
**Independent Variable**	**N**	**%**	**Crude**			**All the Variables Mutually Adjusted**		
			**B**	**SE**	***p***	**B**	**SE**	***p***
Repeated miscarriage								
No	566	89.1	**Referent**			**Referent**		
Yes	69	10.9	**2.48**	**0.71**	**< 0.001**	**1.57**	**0.69**	**0.023**
Intercourse problem								
No	564	88.8	Referent			Referent		
Yes	71	11.2	0.34	0.71	0.628	0.16	0.67	0.807
Sperm problem								
No	474	74.6	Referent			Referent		
Yes	161	25.4	−0.49	0.51	0.338	−0.22	0.49	0.648
Unknown								
No	540	85.0	**Referent**			**Referent**		
Yes	95	15.0	**1.27**	**0.62**	**0.042**	**1.64**	**0.63**	**0.009**
Living with a child or children								
Yes	125	19.7	**Referent**			**Referent**		
No	510	80.3	**2.36**	**0.55**	**<0.001**	**1.65**	**0.58**	**0.005**
Annual income of the couple								
≥80,000 dollars	135	21.3	**Referent**			**Referent**		
≥60,000 and <80,000 dollars	155	24.4	0.11	0.65	0.871	−0.02	0.61	0.979
≥40,000 and <60,000 dollars	228	35.9	**1.83**	**0.60**	**0.002**	**1.29**	**0.59**	**0.029**
<40,000 dollars	117	18.4	**2.37**	**0.70**	**<0.001**	**1.79**	**0.68**	**0.009**
Employment status								
Unemployed	208	32.8	Referent			Referent		
Employed	427	67.2	0.13	0.48	0.781	0.33	0.46	0.469
Opinion about working women								
Women should try to reconcile working and housekeeping/bringing up children	185	29.1	**Referent**			**Referent**		
The woman’s job should not interfere with her household duties and child-care responsibilities	393	61.9	0.46	0.50	0.354	0.13	0.47	0.776
Women should devote themselves to their household duties	21	3.3	**4.14**	**1.29**	**0.001**	**3.29**	**1.21**	**0.007**
Others	36	5.7	−0.40	1.02	0.693	−0.05	0.94	0.961
Having considered stopping treatment								
No	251	39.5	**Referent**			**Referent**		
Yes	384	60.5	**1.82**	**0.45**	**<0.001**	**1.09**	**0.45**	**0.017**
Married life without children is favorable								
Yes	466	73.4	**Referent**			**Referent**		
No	169	26.6	**1.10**	**0.50**	**0.030**	**1.19**	**0.47**	**0.011**
An adoptive relationship would make me insecure								
No	335	52.8	**Referent**			Referent		
Yes	300	47.2	**1.03**	**0.45**	**0.021**	0.68	0.41	0.101
Stressful situations								
High cost of treatments								
No	46	7.2	**Referent**			Referent		
Yes	589	92.8	**2.39**	**0.86**	**0.005**	1.30	0.81	0.107
Insufficient explanation by doctors								
No	509	80.2	**Referent**			**Referent**		
Yes	126	19.8	**2.10**	**0.55**	**<0.001**	**1.36**	**0.52**	**0.009**
Frustration of multiple failed attempts								
No	186	29.3	**Referent**			**Referent**		
Yes	449	70.7	**2.64**	**0.48**	**<0.001**	**1.27**	**0.48**	**0.009**
Differences of opinion with my partner								
No	577	90.9	**Referent**			**Referent**		
Yes	58	9.1	**2.75**	**0.77**	**<0.001**	**2.01**	**0.73**	**0.006**
Strong peer and family pressure								
No	310	48.8	**Referent**			Referent		
Yes	325	51.2	**2.12**	**0.44**	**<0.001**	0.47	0.44	0.293
Feelings of guilt toward my partner								
No	320	50.4	**Referent**			Referent		
Yes	315	49.6	**2.02**	**0.44**	**<0.001**	0.67	0.45	0.135
No idea of when to stop the treatment								
No	300	47.2	**Referent**			**Referent**		
Yes	335	52.8	**2.67**	**0.43**	**< 0.001**	**1.84**	**0.43**	**<0.001**
Physical stresses of the treatment								
No	229	36.1	**Referent**			Referent		
Yes	406	63.9	**1.35**	**0.46**	**0.004**	−0.01	0.47	0.990
Worries about the future health effects of the treatment								
No	369	58.1	**Referent**			Referent		
Yes	266	41.9	**1.61**	**0.45**	**<0.001**	0.57	0.45	0.202

Notes: B = unstandardized regression coefficient; SD = standard deviation; SE = standard error. Bold values signify statistical significance.

However, psychological distress did not seem to affect miscarriage/stillbirth/abortion, cause of fertility, living with or without child, joint income with their partner, and the opinion that “women should devote themselves to their household duties”. Third, although patients from 70 infertility treatment institutions were included, convenience sampling was used and the response rate was low at 25.0%; thus, the results may not be applicable to the entire population of women undergoing infertility treatment. This potential selection bias may have an impact on factors such as the prevalence estimates of psychological distress, although it appears unlikely that it has greatly altered the association of family-related opinions and stressful situations with psychological distress. These results may contribute to the prevention of and care for psychological distress in women undergoing infertility treatment.

## 5. Conclusions

Family-related opinions and stressful situations associated with psychological distress in women undergoing infertility treatment are outlined. The results of this study may contribute to the prevention of and care for psychological distress in female patients undergoing infertility treatment.
